# To Do Or Not To Do: Therapeutic Hypothermia Treatment For An Infant With HIE And Prenatal Spinal Muscular Atrophy With Congenital Bone Fractures

**DOI:** 10.34763/jmotherandchild.20263001.d-25-00033

**Published:** 2026-03-06

**Authors:** Viktoryia Parfenchyk, Mateusz Jagła

**Affiliations:** Department of Pediatrics, Jagiellonian University Medical College, Kraków, Poland

**Keywords:** therapeutic hypothermia, spinal muscular atrophy

## Abstract

Spinal muscular atrophy with congenital bone fractures is a rare, severe neuromuscular disorder with autosomal recessive inheritance, characterised by hypotonia, congenital contractures, and respiratory distress. We present the case of a newborn girl with a homozygous mutation in the ASCC1 gene, who was diagnosed with hypoxic-ischaemic encephalopathy after birth and underwent therapeutic hypothermia (TH). Although TH did not cause any side effects, it also did not improve the prognosis or quality of life of the patient. The decision whether to perform TH in neonates with congenital or genetic abnormalities remains challenging. Current exclusion criteria for TH should be re-evaluated to support clinicians in determining whether to include newborns with severe congenital abnormalities but favourable neurological prognosis, and conversely, to exclude those with congenital or suspected genetic syndromes associated with poor life expectancy and quality of life, in order to avoid futile interventions.

## Introduction

Spinal muscular atrophy with congenital bone fractures is a rare, severe neuromuscular disorder with autosomal recessive inheritance. The clinical presentation typically includes foetal hypokinesia, severe hypotonia, congenital contractures, respiratory distress, lung hypoplasia, and prenatal fractures of long bones (like the femur and humerus). The prognosis is poor, with life expectancy ranging from a few days to a few months.

Recent data indicate that spinal muscular atrophy with congenital bone fractures is caused by homozygous or compound heterozygous mutations in the *ASCC1* gene located on chromosome 10q22 [ 1,2,3,4].

We present the case of a newborn girl with a homozygous mutation in the *ASCC1* gene, who was diagnosed with hypoxic-ischaemic encephalopathy after birth and underwent therapeutic hypothermia. We aim to discuss whether the use of therapeutic hypothermia was justified in this clinical context.

## Case Presentation

A female neonate was delivered via caesarean section at 37 weeks of gestation due to breech presentation and suspected intrauterine growth restriction. Birth weight was 2400 g, body length was 54 cm, and head circumference was 34 cm. Apgar scores were 2, 4, 4, and 4 at 1, 3, 5, and 10 minutes, respectively. At birth, the neonate was flaccid, pale, unresponsive, and apnoeic. Positive-pressure inflation breaths were administered, followed by positive-pressure ventilation. After transferring to the neonatal intensive care unit (NICU), the infant was intubated. A blood gas analysis performed within the first hour of life revealed metabolic acidosis (pH 6.99, base excess −12.7). The Thompson score was 13 points [5]. Physical examination revealed a fracture of the left humerus and numerous petechiae.

The obstetric history was unremarkable. The parents’ first child was healthy.

Given the low Apgar scores, metabolic acidosis, and clinical signs of severe hypoxic-ischaemic encephalopathy (HIE), the neonate qualified for therapeutic hypothermia (TH) and was transferred by medical transport at 4 hours of life to a tertiary NICU where TH could be initiated.

On admission, the infant was in critical condition— intubated, mechanically ventilated, hypotensive, and sedated with ketamine. The child was unresponsive. Dysmorphic features and generalized hypotonia were observed. The skin appeared pale and mottled, with increased turgor and multiple petechiae and bruises. Swelling of the left upper limb and subperiosteal haematomas on the head were noted.

Laboratory findings revealed leucocytosis with neutrophilia, mild microcytic anaemia, elevated lactate, hypoalbuminaemia, hyperglycaemia, impaired coagulation, and increased cardiac markers (troponin T and NT-proBNP). Blood gas analysis after admission showed metabolic acidosis (pH 7.28, BE −7.6). Blood, tracheal aspirate, and stool cultures taken at admission were sterile. Congenital cytomegalovirus infection was excluded.

**Figure 1.**
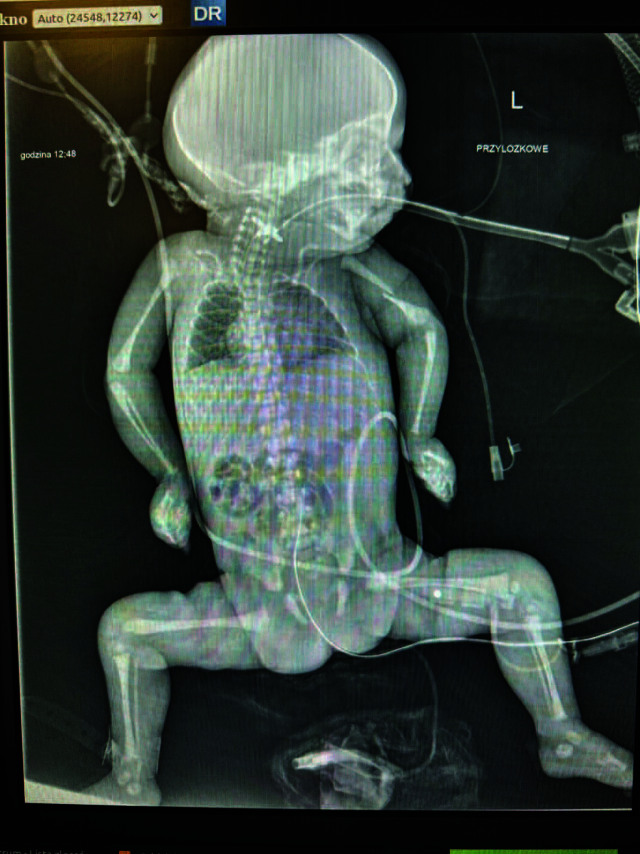
Babygram of the patient.

A cranial ultrasound revealed a right choroid plexus cyst, widened interhemispheric fissure, suspected fracture of the left humerus, and possible pulmonary hypoplasia. A skeletal survey confirmed the humeral fracture (Figure 1).

The neonate was qualified for whole-body therapeutic hypothermia. During the procedure, the infant required circulatory support with dopamine, diuretic stimulation, and hydrocortisone. Due to hyperglycaemia, insulin infusion was administered for one day. The patient also required transfusions of cryoprecipitate and fresh frozen plasma.

On day 5 of life, brain MRI with spectroscopy demonstrated hypoxic-ischaemic changes in both cerebral hemispheres, the left thalamus, corpus callosum, and—less prominently— the cerebellar hemispheres. Findings also included post-intraventricular haemorrhage and a post-dural haematoma in the right cerebellar hemisphere. The lactate/N-acetylaspartate (Lac/NAA) ratio was 0.1606 in the right and 0.2590 in the left thalamus (Table 1).

**Table 1.** Results of MRI spectroscopy.

**Localization VOX**	**Volume VOX (cm^3^)**	**Lac / NAA**	**Lip / Cr**	**NAA / Cr**	**GABA / Cr**	**Cho / Cr**	**Cho / NAA**	**Lac / Cr**	**GLN / Cr**
Right thalamus	1,69	0,1606	0,6711	1,0361	0,4400	1,1940	1,1524	0,1664	0,3509
Left thalamus	1,69	0,2590	0,1863	0,8733	0,2849	1,4509	1,6615	0,2262	0,1729

On day 12 of life, Klebsiella pneumoniae (ESBL strain) sepsis was diagnosed. Lumbar puncture confirmed central nervous system infection. Despite targeted antibiotic therapy, cultures remained positive for an extended period.

During hospitalization, the patient developed pneumonia three times, caused by *Staphylococcus haemolyticus*, *Stenotrophomonas maltophilia*, and *Klebsiella pneumoniae* (ESBL strain).

On day 9 of life, ultrasound revealed bilateral pleural effusions, requiring drainage. Biochemical analysis of the fluid confirmed chylothorax. Cultures were sterile. Enteral feeding was discontinued, and somatostatin infusion was initiated. Despite therapy, chylothorax persisted. Due to the patient’s deteriorating condition and poor prognosis, surgical management was not pursued.

Throughout hospitalization, the neonate exhibited persistent generalized oedema. Diuretics (furosemide and continuous infusion of bumetanide) were ineffective.

Despite sedation reduction, the infant remained apnoeic and showed minimal spontaneous activity limited to slight movements of the extremities and face.

Neurological examination in the second week revealed an unresponsive infant with an amimic face, abducted– semi-extended limb positioning, and pronounced oedema. The skin was taut, with extensive bruising and flexion contractures of the fingers. Deep tendon reflexes and primitive reflexes were absent.

A genetic consultation on day 5 raised suspicion of a congenital connective tissue disorder and whole exome sequencing (WES) was recommended. Spinal muscular atrophy was excluded. Chromosomal microarray analysis (CNV) was normal.

WES with CNV analysis revealed a homozygous deletion in chr10:(72213298–72210731)x0, corresponding to exons 2–3 of the *ASCC1* gene (NM_001198800.3). Similar variants are classified as pathogenic in ClinVar (IDs: 1460373, 1210120). Runs of homozygosity (ROH) analysis showed polymorphic variants in the 10q22.1 region (containing *ASCC1*) occurring only in homozygosity, suggesting potential uniparental isodisomy. Pathogenic *ASCC1* variants are associated with prenatal spinal muscular atrophy with congenital bone fractures (OMIM: 616867).

In the 8th week of life, due to progressive clinical deterioration, extremely poor prognosis, and lack of therapeutic options, a multidisciplinary team initiated a palliative care protocol. Management included analgesic sedation, mechanical ventilation, and parenteral nutrition. Eleven days later, the infant experienced cardiac arrest and was pronounced dead.

Post-mortem examination revealed generalized ischaemic encephalopathy with oedema and secondary hyperaemia. Focal fibrosis was observed in the myocardium of both ventricles and the interventricular septum. The lungs were mildly hyperaemic with macrophage clusters in the alveoli and focal epidermal desquamation. The liver exhibited bile duct proliferation, small chronic inflammatory infiltrates, and intensified cholestasis. Fluid was present in both pleural cavities and the peritoneal cavity.

## Discussion

Hypoxic-ischaemic encephalopathy (HIE) is one of the leading causes of neurological disability in children, occurring in 1–2 per 1,000 live births [6]. Therapeutic hypothermia (TH) is currently the only evidence-based intervention for HIE shown to reduce neurological disability and the incidence of cerebral palsy during infancy and early childhood. However, its effect on mortality remains inconclusive [7]. Among the exclusion criteria for TH are the presence of major congenital anomalies or abnormalities suggestive of chromosomal or genetic syndromes [8,9].

These exclusion criteria leave the decision to the attending neonatologist whether or not to qualify an infant with suspected or confirmed congenital or genetic anomalies for TH. This decision is further complicated by the narrow therapeutic window of six hours after birth, which precludes the use of genetic testing or other time-consuming diagnostic methods before the initiation of TH.

In our case, dysmorphic features, severe hypotonia, areflexia, and a humeral fracture could have indicated an underlying genetic disorder. However, it was not possible to distinguish whether these symptoms were due to HIE or a congenital condition at the time. Therefore, TH was initiated. Although no adverse effects were observed, TH did not improve the prognosis or quality of life in this patient. The result of whole exome sequencing (WES), which confirmed the diagnosis of spinal muscular atrophy with congenital bone fractures, became available only after the patient’s death and thus could not contribute to treatment decisions. This raises the question of whether TH constituted a futile intervention in this case.

Nonetheless, despite the lack of benefit in our patient, the broader question of whether to apply TH in neonates with congenital or genetic abnormalities remains complex.

The literature provides examples of infants who received TH despite congenital anomalies. For instance, one case report describes an infant with HIE and gastroschisis who required prolonged resuscitation at birth and underwent TH. On day 8 of life, the abdominal wall defect was surgically closed. At six months of age, developmental assessment using the Bayley Scales of Infant and Toddler Development – Fourth Edition (Bayley-4) revealed only mild receptive and expressive language delay and mild gross motor delay [10].

Another report describes two cases of infants with a congenital diaphragmatic hernia and HIE who were treated with TH. Surgical repair of the diaphragm was performed on days 2 and 3 of life. The authors did not observe the exacerbation of pulmonary hypertension or surgical complications. Brain MRI at one month revealed no abnormalities, and Bayley-III scores at 18 months were within average and low-average ranges [11].

There is no consensus regarding the use of TH in neonates with congenital heart defects (CHD) and HIE. A survey of tertiary neonatal departments in Germany revealed significant variability in clinical practice. While simple CHD had little influence on the decision to initiate TH, more complex defects raised more concern, despite limited evidence [12].

Some authors advocate the use of TH in patients with severe CHD and HIE. One case report and a retrospective observational study suggest that TH does not compromise the efficacy of low-dose prostaglandin E1 (PGE1) in ductal-dependent CHD, nor the safety of subsequent cardiac surgery [13,14]. However, adverse effects are common and should be carefully considered, as TH may be associated with increased risk of haemodynamic instability.

Not all outcomes in infants with congenital anomalies receiving TH are favourable. A study by Mrelashvili et al. reported on eight infants with syndromic disorders or congenital anomalies (categorized into three groups: dysmorphic craniofacial features without identified aetiology, isolated brain malformations, and recognized syndromes) who underwent TH for HIE. The results were discouraging. Mortality was high, and among the four survivors assessed at a median age of 26 months, three had poor neurodevelopmental outcomes [15].

It is conceivable that the exclusion criteria for TH should be revised to guide clinicians in qualifying neonates with severe congenital anomalies who may still have favourable neurological prognoses, and to disqualify those with poor expected life quality and prognosis, in order to avoid futile interventions.

## Conclusion

There is significant variability in clinical practice regarding the disqualification of neonates from therapeutic hypothermia. Unfortunately, due to the very limited number of newborns with hypoxic-ischaemic encephalopathy (HIE) and coexisting congenital or genetic abnormalities, no clinical trials have been conducted to standardize management in such cases — and it is unlikely that such trials will be feasible in the future. Consequently, each case must be assessed on an individual basis. Moreover, the diagnostic process in neonates with suspected genetic conditions is often complex and time-consuming, while decisions regarding qualification for therapeutic hypothermia (TH) must be made within a narrow time window. As a result, similar cases to the one presented here are likely to occur in the future.
